# Nanotechnology-Related Environment, Health, and Safety Research: Examining the National Strategy

**DOI:** 10.1289/ehp.117-a158

**Published:** 2009-04

**Authors:** Charles W. Schmidt

Pick up a tube of sunscreen, a tennis racquet, an iPod, or any number of other consumer products, and there’s a good chance that it’s been “nano-enabled,” meaning it contains nanoscale particles designed to give it some beneficial feature. An estimated $147 billion worth of nano-enabled commercial and consumer products were sold in 2007, according to Lux Research, a market analysis firm in New York City. Citing the firm’s latest estimates, Lux analyst David Hwang predicts that figure could top $3.1 trillion by 2015, reinforcing a broad view that nanotechnology is fueling a new industrial revolution.

Yet nanotechnology’s spread through the market has been met with mounting concerns over the potential human health effects of these miraculous materials. Because of their small size—100 nano-meters or less—nanomaterials have unique physical properties that can influence their uptake, distribution, and behavior in the body. Indeed, some nano-particles have been shown to penetrate into cells, where they can trigger inflammatory responses and oxidative stress.

Canada and California recently took the unprecedented step of imposing mandated disclosure requirements on nanomaterial use and toxicity assessment. Issued 29 January 2009, Canada’s law targets domestic companies and institutions that manufacture or buy more than 1 kilogram of nanomaterial per year. According to the new regulations, these entities must now reveal how much nanomaterial they use, how they use it, and what they know about its toxicity. California’s law, issued 2 February 2009, limits its scope to carbon nanotubes, a class of nanomaterial used in electronics, optics, and biomedical applications. Under the new regulation, by February 2010 companies that manufacture, import, or export carbon nanotubes in California must disclose information about the toxicity and environmental impacts of their products.

Meanwhile, experts in nanotoxicology and risk assessment have become increasingly polarized, represented on one side by the National Research Council (NRC) and on the other by the National Nanotechnology Initiative (NNI), a government-wide collaboration coordinated by the National Science and Technology Council in the Executive Office of the President. In February 2008, the Nanotechnology Environmental and Health Implications (NEHI) Working Group of the NNI released a document titled *Strategy for Nanotechnology-Related Environmental, Health, and Safety Research*. This document is meant to present the U.S. government’s agenda for studying nano-particle hazards, and describes 246 related projects that were ongoing in 2006, representing a combined investment for that year of $68 million. The document also purports to “address prioritized research areas . . . and to advance knowledge and support risk decision-making—both of which are essential for the responsible development of nanotechnology.”

Clayton Teague directs the National Nano-technology Coordination Office, which was responsible for drafting the federal strategy. He says the strategy was developed in extensive consultation with regulatory agencies, research organizations, the business community, and nongovernmental organizations. “We believe the strategy represents needs and agreements about what the agencies plan to do,” he says. “Funding agencies are telling us that they’re using the document to formulate solicitations for future research in this area.”

But on 25 February 2009, a panel assembled by the NRC issued its own report, describing what it calls serious shortcomings in the strategy document. According to the NRC panel, which was assembled at the request of the NNI, the strategy exposes weaknesses in the government’s understanding of potential nanotechnology risks today and does not adequately address how they will be assessed in the future. NRC panel member Mark Weisner, a professor of civil and environmental engineering at Duke University, claims that many of the research programs described in the NNI’s document don’t actually address environmental, health, and safety (EHS) concerns. “If you take this portfolio at face value, it overstates the true level of effort in federally financed [nano-technology-related] EHS research,” he says.

## Toxicity Unknowns

No case of human toxicity has been linked to the roughly 2,000 types of nano-materials in commercial use or development today. Yet those risks can’t be ruled out, says Günter Oberdörster, a professor of environmental medicine at the University of Rochester School of Medicine and Dentistry. According to Oberdörster, multiwalled carbon nanotubes have been found to elicit responses similar to those seen with fibers of chrysotile asbestos, a known human carcinogen. Oberdörster emphasizes these findings have been seen only in rodents given carbon nanotubes at extremely high doses by injection. What’s needed now, he says, are toxicity data generated by inhalation routes that mimic real-life human exposure. Oberdörster says he and others in the field are currently working on such studies.

Predictions about nanotechnology risk have emerged from inhalation research, specifically studies targeting ultrafine soot particles with nanoscale dimensions. Upon inhalation, some of these particles traverse epithelial and endothelial cells to reach the blood and lymph circulation, which carries them to potentially sensitive sites, including the bone marrow, lymph nodes, spleen, heart, and central nervous system. *In vitro* and animal studies show these particles can—depending on the dose and chemical composition—induce a range of inflammatory effects, whereas epidemiologic findings link them to respiratory and cardiovascular diseases.

All nanoparticles have high surface-to-mass ratios, which makes them uniquely reactive in the body. “Chemical reactions tend to occur at particle surfaces,” explains Jeff Morris, associate director for science in the U.S. Environmental Protection Agency (EPA) Office of Science Policy. “Given that their surface area exceeds their mass, nano-particles tend to be more reactive than larger particles with the same chemical makeup.”

Engineered nanoparticles and soot differ in key ways, however. In particular, soot is heterogeneous in terms of particle size, chemistry, surface characteristics, and other constituents, whereas engineered nano-particles—within product categories—have uniformly identical shapes, including spheres, tubes, wires, rings, and planes. Given their similar high surface-to-volume ratios, both types of particles could trigger comparable biologic effects, Oberdörster adds.

But particle uniformity might also influence the kinetics and toxicity of nano-materials in unknown ways. For instance, Andrew Maynard, science advisor to The Project on Emerging Nanotechnologies, a collaboration of the Pew Charitable Trust and the Woodrow Wilson Center for International Scholars, proposes that some of the particles in soot and other heterogeneous mixtures could be more harmful than others. “In that case, the toxicity of the harmful particles is diluted by the presence of others that are less so,” he explains. “But when you engineer particles with precise characteristics, you lose that dilution factor, and the chance of producing something uniformly dangerous increases.”

## Looking for a Strategy

The NRC panelists would like to see a national, health-based strategy for nanotech-nology research, with defined goals, milestones, and mechanisms for assessing progress. Maynard stresses the need isn’t just to ensure the safety of nano-enabled products, but also to avert a public backlash against the technology, which could grow if health risks aren’t seen to be adequately addressed. Yet the NNI strategy document—NRC panelists claim—is simply a compendium of federally funded projects without any unifying vision or sense of shared purpose. Each of the projects listed by the NNI is grouped under one of five research categories: instrumentation, metrology, and analytical methods; nanomaterials and human health; nanomaterials and the environment; human and environmental exposure assessment; and risk management methods.

In Maynard’s view, these projects aren’t adequately organized around questions of public concern. Instead, they reflect investigator-motivated studies, piqued by each scientist’s own interests, he asserts. “Scientists don’t like being told what to do,” Maynard acknowledges. “But there’s a disconnect between what might interest them and what companies and regulators who deal with nanotechnology actually need.”

Sally Tinkle, senior science advisor in the NIEHS Office of the Director and cochair of the Nanoscale Science, Engineering, and Technology (NSET) subcommittee of the National Science and Technology Council, says federal agencies have had to make do without any federal appropriation specifically for nanotoxicology research. “The agencies have to fund what they can under flat budgets with competing research priorities,” she says.

A prepublication copy of the NRC report was leaked to the press 10 December 2008. The ensuing media attention prompted the NNI on 5 January 2009 to post an 18-page rebuttal on its website, http://www.nano.gov/, which stated that the strategy document is not, and was never intended to be, a strategic plan or an implementation plan, but rather a higher-level description of the interagency approach to nanotechnology-related EHS research; “[i]t was written as a strategy document for federal agencies in order to coordinate, encourage cooperation, and where possible to implement collaborative research actitivies.” The rebuttal goes on to list what it calls technical errors in the NRC assessment. Although such errors were corrected in the final February 2009 release, the NRC did not change its overall conclusions.

Of paramount importance, Wiesner says, is that exposure and toxicity research in nanotechnology be balanced appropriately. “We don’t want the toxicity work to get too far out in front of the exposure work, and yet the toxicity work tells us where we should focus our exposure studies,” he says. “There’s a delicate balance we need to achieve here, and that’s something the whole research community is struggling with right now.”

Exposure research in nanotechnology does come with unique challenges, Wiesner acknowledges. Scientists have yet to develop widely accepted methods for introducing nanomaterials into living systems such as cell cultures, for instance. As nanomaterial surfaces interact with cell macromolecules and salts, their properties can change in mysterious ways. And those transformations, Wiesner says, directly influence interpretations of effective exposure and dose response.

## Questions Regarding Transparency

Meanwhile, given mounting scrutiny, industry has become more sensitive about its public image vis-à-vis nanomaterials, according to Ellen Kenney, a senior research analyst with the Bethesda investment firm Calvert Group, Ltd. Companies that don’t use nano-materials have begun stating so in their shareholder reports, she says, in a reflection of how these materials might be viewed as a public liability.

Indeed, many companies are reluctant to reveal their nanomaterial use and toxicity data voluntarily. With its Nanoscale Materials Stewardship Program (NMSP), launched 28 January 2008, the EPA urges companies to report available information about the engineered nanoscale materials they manufacture, import, process, or use. This voluntary two-year effort is intended to help inform eventual regulatory decisions about nanomaterials. According to Lux Research, the total number of companies engaged in nanotech production or use could reach 1,000. The EPA reached out to more than 150 companies and 11 trade associations, Hwang says, but by the time the NMSP published its interim report on 12 January 2009, only 29 companies had responded. In total, these companies disclosed data on 123 nanomaterial compounds.

Jim Willis, who directs the EPA Chemical Control Division, says those results left agency personnel with mixed feelings. “On the one hand, we thought it was pretty good responsiveness for a volunteer program,” he says. “On the other, we know there are hundreds of other nanomaterials that weren’t reported. And that indicates clearly that we need to do more if we want to get a better handle on what’s being produced, at what levels, and how humans are being exposed.”

Sources interviewed for this article unanimously agreed that nanomaterials promise valuable benefits for society, among them better drugs; stronger, lighter products; and better environmental and energy technologies. But nanoparticle toxicity data need to be made more widely available to ensure public support for the technology. Jennifer Sass, a staff scientist with the Natural Resources Defense Council, says such data typically wind up in company reports instead of in publicly available, peer-reviewed research journals. And Julia Moore, deputy director of The Project on Emerging Nanotechnology, claims the public has limited access to information about which companies use nanomaterials and how. “That information isn’t in the hands of government regulators,” she says. “It’s in the hands of market analysts on Wall Street, and they’re not going to let it go without a price.”

Those on the industry side believe many interest groups have significantly overhyped the dangers of nanomaterials. “Fear-mongering both inhibits industry efforts to encourage companies utilizing nanotechnology to do so in a visible way by ‘branding’ it and makes it more difficult for entrepreneurs to raise capital and find partners to bring new innovations to market,” says Sean Murdoch, executive director of the Nanobusiness Alliance, a trade group based in Skokie, Illinois. He cites the comparison between carbon nanotubes and chrysotile asbestos as an example. He points out that, unlike asbestos, which for decades was mined at million-ton quantities by unprotected workers, carbon nanotubes are processed in laboratories subject to strict safety protocols. Moreover, he says, the nanotubes themselves, once incorporated into products, have no bioavailability. “There’s no way the exposure scenarios are comparable,” he says.

But this argument does not address product end-of-life concerns, say many experts. Tinkle says, “There is still concern over exposure to nanoparticles at the end of the products’ life cycles, even if companies design the product to be completely safe for the immediate user. Once [a nano-enabled item] is thrown out and begins to decompose or degrade—or it begins to break down from day-to-day use—the particles can be released into the environment. Care needs to be taken to control the exposure throughout the product life cycle.”

For his part, Oberdörster suggests most nanoparticles may turn out to be benign under real-life exposure conditions. “I think there’s a certain amount of hype surrounding the toxicity issues,” he says. “However, until we know better, we should be careful and avoid exposure. You can do a lot of *in vitro* testing at high doses and identify a hazard, but you need the necessary exposure for a risk to be present.”

Still, assuming the growth trends continue, nanomaterials will be produced at ever-increasing quantities, and public and environmental exposures will rise commensurately. Given that reality, the industry’s future may well rest on its transparency to public scrutiny.

## Figures and Tables

**Figure:**
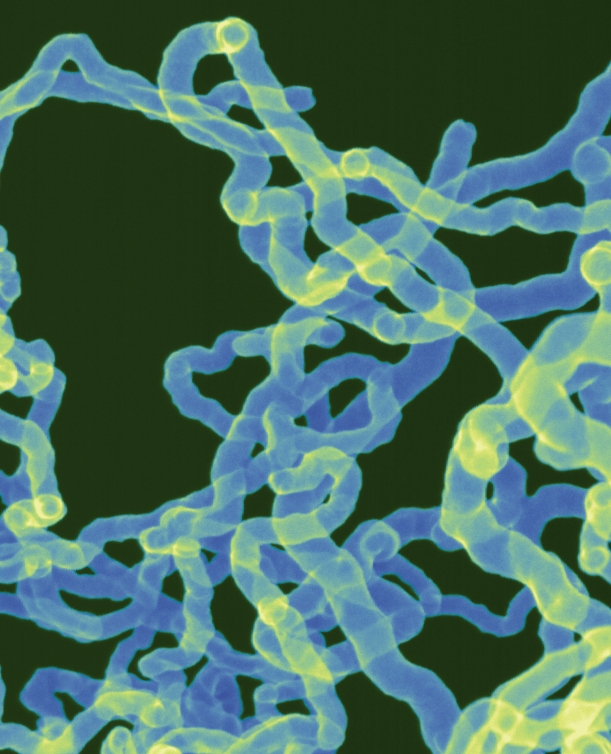
Scanning electron micrograph of carbon nanotubes, magnified 40,000 times.

**Figure:**
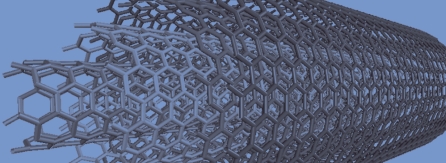
The NEHI Working Group developed this . . . research strategy to accelerate progress in research to protect public health and the environment, and to fill gaps in, and—with the growing level of effort worldwide—to avoid unnecessary duplication of, such research. — NNI Strategy Document

**Figure:**
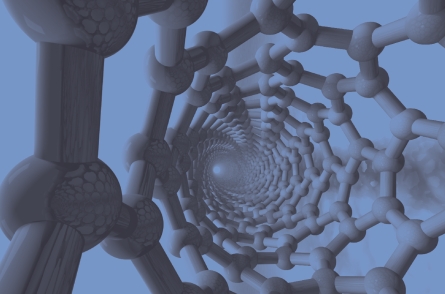
The process of composing the government’s 2008 NNI document provided a unique and useful opportunity for coordination, planning, and consensus-building among NEHI-member federal agencies. . . . However, [the document] does not have the essential elements of a research strategy—it does not present a vision, contain a clear set of goals, have a plan of action for how the goals are to be achieved, or describe mechanisms to review and evaluate funded research and assess whether progress has been achieved in the context of what we know about the potential EHS risks posed by nanotechnology. —NRC report

